# Emotional responses to art *in situ* and on screen: a test of the circumplex model and the genuineness effect

**DOI:** 10.3389/fpsyg.2026.1960728

**Published:** 2026-10-08

**Authors:** Cindy Chamberland, Valentin Perichon, Sébastien Tremblay

**Affiliations:** School of Psychology, Université Laval, Quebec City, QC, Canada

**Keywords:** circumplex model, emotion, empirical aesthetics, genuineness effect, museum studies

## Abstract

**Introduction:**

Emotional responses are commonly measured within the valence–arousal dimensional space of Russell’s (1980) circumplex model, although the extent to which this organization captures emotional responses to art remains debated. We examined the correspondence between art-elicited emotional responses and this two-dimensional organization. We compared the correspondence in two viewing contexts and used the same representational space to assess the genuineness effect, that is, the difference in experience between an original artwork and a digital reproduction.

**Methods:**

Two groups of 52 participants viewed the same 17 artworks during a visit to the Musée national des beaux-arts du Québec (genuine) or in the laboratory (digital format). Following each artwork consultation, participants first selected their main emotion from a set of predefined emotions and subsequently rated valence and arousal using visual analog scales on an iPad application.

**Results:**

Observed emotion centroids showed correspondence with the external valence–arousal reference structure in both contexts, with similar patterns of correspondence for the museum and the laboratory. Fascination emerged as the most frequently reported emotion, and 7% of responses fell outside the predefined set of emotions derived from dimensional models. No significant differences were found between the museum and laboratory contexts for valence, arousal, or viewing time.

**Discussion:**

Our findings indicate that emotional responses to artwork correspond closely to the dimensional organization of valence and arousal at the level of emotion centroids. However, the emotion categories derived from that organization do not fully capture what viewers report. That originals and digital reproductions elicited comparable emotional responses further suggests that the genuineness effect is either absent or very small.

## 1 Introduction

Emotion has come to be seen as the central outcome of the art experience rather than as a by-product of aesthetic judgment (Funch, 2022; Tinio and Specker, 2023). Describing it, however, requires a frame of reference, and in empirical aesthetics that frame is most often the valence–arousal space proposed by Russell (1980). Whether this representational space is adequate for art is contested (e.g., Fontaine et al., 2007; Marković, 2012; Schindler et al., 2017). This question is descriptive and methodological: it concerns whether the valence–arousal space, and the emotion lexicon built upon it, capture what viewers report in front of artworks, and whether they behave equivalently across viewing conditions. This second point matters for the genuineness effect (that is, the difference in experience between an original artwork and a digital reproduction; Brieber et al., 2015a), which meta-analytic evidence suggests is small and heterogeneous (Specker et al., 2023). The present study, therefore, examines the correspondence between the emotions viewers report and their reference coordinates in the circumplex model for artworks encountered both as genuine in a museum and as digital reproductions in the laboratory, and uses the representational space of the circumplex model to test whether genuineness modulates emotional response.

### 1.1 The nature of emotional response to artwork

Russell’s (1980) circumplex model is among the most widely used frameworks for describing emotional experience (see Shuman et al., 2013). Rather than positing a set of discrete, biologically given categories (cf. Cowen and Keltner, 2017), it locates affective states within a two-dimensional space defined by valence (pleasure vs. displeasure) and arousal (activation vs. deactivation), with emotion terms distributed around the circumference of a circle. Emotional states adjacent on the circle are experientially similar, whereas opposing states are antithetical. Russell and Barrett (1999) later specified that the model describes core affect, that is, continuous, object-free states of pleasure and activation, rather than providing an exhaustive taxonomy of emotions, and conceived valence as a single bipolar dimension, an assumption that remains debated (see Walle and Dukes, 2023). Subsequent psychometric work refined the resolution of this space: Yik et al. (2011) proposed a 12-point circumplex integrating the major dimensional models of mood and emotion, offering finer coverage than earlier quadrant-level solutions.

The valence–arousal space has been applied across music, language, consumer experience, and psychophysiology, and in most of these domains the two-dimensional structure is reliably observed. In music, Eerola and Vuoskoski (2011) compared discrete and dimensional descriptions of emotion across 110 film-music excerpts and found convergence along two dimensions interpretable as valence and arousal, with the dimensional account outperforming the categorical one for emotionally ambiguous material; the same dimensions are used in computational models of music emotion recognition (Griffiths et al., 2021). In consumer research, satisfaction has been decomposed into valence- and arousal-like components associated with behavioral intentions (White and Yu, 2005). In psychophysiology, Russell’s model serves as the reference frame for estimating affective state from peripheral signals (Cittadini et al., 2023). In language, Paltoglou and Thelwall (2013) derived valence and arousal norms for naturally occurring text; these norms provide an external reference structure for the valence–arousal organization of emotion terms against which the observed responses are compared in the present study.

Whether dimensional models of everyday emotions, such as the circumplex model, can fully capture aesthetic experience remains an open question. Russell’s circumplex is the most widely used of these models, though others propose additional dimensions such as power and novelty (Fontaine et al., 2007). On one side, the terms viewers use in front of artworks include states (fascination, being moved, and awe) that are poorly represented in the circumplex lexicon and whose valence is often mixed rather than simply positive or negative (Schindler et al., 2017). When art-elicited experience is mapped without applying a small set of *a priori* labels, it proves richer than two dimensions: Stamkou et al. (2024) collected reports on 1,457 artworks and recovered 25 non-reducible categories, including emotions (e.g., mysterious, spiritual) absent from both the standard emotion lists. On the other side, art-elicited emotions have been reported to map onto valence and arousal (Tinio and Gartus, 2018; Tschacher et al., 2012), and Skov and Nadal (2020) argue that there is no evidence for emotional processes specific to art: affective responses to artworks are generated by the same valuation and reward mechanisms that process food, music, or monetary gain, and no dedicated neural system for aesthetic emotions has been identified. Based on this account, what distinguishes the art context is the object and the framing of the encounter, not the emotional processing it involves (see also Fingerhut and Prinz, 2020). If this is correct, emotions reported in front of artworks should adhere to the same dimensional structure documented in other domains.

A further consideration is that evidence for the applicability of the circumplex model in visual affective research is largely based on photographs presented on screens (e.g., Cittadini et al., 2023), a limitation of context-free static stimuli raised by Walle and Dukes (2023). Whether the model can represent emotional responses to original artworks encountered in a museum has rarely been tested. The first aim of the present study is therefore to assess the correspondence between where viewers place themselves in valence–arousal space and where the emotion they name is theoretically located, in both a museum and a laboratory.

### 1.2 The genuineness effect and emotional response

Meta-analytic evidence suggests that the magnitude of the genuineness effect is rather modest, and results across studies are heterogeneous (Specker et al., 2023). A first source of variability seems to lie in the choice of dependent variables. Much of the earlier work has mainly relied on hedonic evaluations such as liking, beauty, or preference (Leder et al., 2004; Locher and Dolese, 2004; see for a review, Nadal and Vartanian, 2022), while other studies have also included behavioral markers such as viewing time (Brieber et al., 2014) or psychophysiological indices (Gerger et al., 2014). Specker et al. (2023) argue that focusing on hedonic outcomes may hide finer-grained emotional dynamics, potentially underestimating genuineness-related differences. When emotional responses are assessed directly, the genuineness effect appears more pronounced. Siri et al. (2018), for instance, reported that genuine artworks elicited stronger emotional responses (both in self-reported involvement and physiological measures of arousal such as skin conductance) than high-quality reproductions, despite no differences in ratings of color intensity or overall aesthetic evaluation (see also Szubielska and Imbir, 2021, who observed an effect of physical context on measures of emotional responses). Another factor reported in meta-analyses is the number of artworks included in an experimental sequence (Specker et al., 2023). Larger sets can lead to “museum fatigue,” reducing engagement as visitors progress (van Paasschen et al., 2015).

A growing body of research shows that where and how artworks are presented modulates responses (see for a review Specker et al., 2023). In most experiments on the genuineness effect, genuine works were viewed in a museum while reproductions were presented in a laboratory setting, making it difficult to attribute differences in experience to the artwork itself rather than to the surrounding environment (Brieber et al., 2014). There is empirical evidence that when context is controlled, the genuineness effect largely disappears (Brieber et al., 2015a). This suggests that the museum setting – with its architecture and curatorial framing – plays a dominant role in shaping aesthetic experience. Indeed, Specker et al. (2017) revealed that museum visitors not only reported richer aesthetic experiences than laboratory participants but also showed context-dependent differences in memory and interpretation, with museums scaffolding deeper engagement through visual detail and contextual information. Darda et al. (2025) extend this conclusion: Even though liking and beauty judgments were similar across museum and digital settings, participants reported greater understanding when viewing artworks in museums, which can be seen as highlighting the reflective outcomes as a distinctive contribution of the museum context.

The effect of genuineness is very difficult to disentangle from the institutional and social contexts in which artworks are encountered (though Brieber et al., 2015a; Grüner et al., 2019, who conceived fully factorial designs, have shown that genuineness effects disappear once context is controlled). Studies conducted in laboratory conditions with digital stimuli may underestimate the impact of genuineness because they lack the ritual, environmental, and embodied dimensions of the museum setting (Benford et al., 2022). Brieber et al. (2014) interpreted the additional time spent with genuine artwork in the museum as reflecting the heightened cognitive–emotional involvement afforded by originals in their natural context (see also Scherer and Moors, 2019). While this finding highlights the value of behavioral markers as indirect indicators of emotional engagement, the present study extends this approach by examining emotional responses more systematically as a means of characterizing the art experience with genuine artwork viewed in the museum vs. that related to reproductions of the same artwork viewed in the laboratory.

### 1.3 The present study

The present study investigates how well emotions elicited by artwork map onto the bidimensional structure of the circumplex. We also tested the genuineness effect in visual art using emotional response as the central dependent variable rather than preference or general liking. In collaboration with the Musée national des beaux-arts du Québec (MNBAQ), participants were exposed to the same set of artworks in both genuine (museum) and digital (laboratory) contexts. We are interested in the overall, ecologically valid impact of viewing genuine artworks *in situ* (that is, originals presented within the natural, multisensory, and curated environment of the museum) compared with viewing their digital reproductions in a laboratory-like online setting (see Tröndle et al., 2022). This combination of genuineness and physical context reflects how artworks are typically encountered in real life: museums present originals, while digital reproductions circulate outside of them. The novelty of our approach lies in assessing this combined impact with more direct and systematic measures of emotional response, extending beyond hedonic evaluations of liking or preference.

Our approach is positioned at the intersection of cognitive psychology and empirical aesthetics. From a cognitive and neuroaesthetic perspective, art experience involves perceptual, evaluative, and emotional processes that unfold within a broader contextual framework (Chatterjee and Vartanian, 2014). At the same time, empirical aesthetics emphasizes the importance of investigating art experiences with ecologically valid (especially in naturalistic settings such as museums; Benford et al., 2022; Tröndle et al., 2011) and interdisciplinary methods (Specker, 2025; Tröndle et al., 2022). Our design follows this call by directly comparing artworks in a museum setting with their high-quality digital reproductions in a laboratory, while placing emotional responses as the primary outcome (Brieber et al., 2015b; Tinio and Specker, 2023).

Emotional experience was measured along the valence–arousal dimensions (measured with digital scales; Betella and Verschure, 2016) as well as through categorical emotion labels. This approach allows us to address two questions. First, do participants’ reported emotions align with Russell’s (1980) circumplex model, and does the emotion lexicon that can be derived from that model cover the emotions viewers report? Second, do genuine artworks viewed *in situ* elicit stronger and more polarized emotions than their digital reproductions viewed outside the museum, thereby extending the genuineness effect beyond preference into the affective domain? A step toward measuring emotional responses more directly was taken by Siri et al. (2018). While their study suggests that genuineness effects become more visible when emotional response is measured, their assessment of emotion was relatively broad (a single self-report item of emotional involvement, “How emotionally involved were you?” on a Likert scale, and two autonomic indices of arousal: skin conductance and heart rate). In the present study, we extend this line of research by assessing emotional responses more systematically.

In our design, one group of participants encountered high-quality reproductions of the artwork (paintings, sculptures, and compositions) and one group viewed the originals in the MNBAQ. Special attention was given to the quality and resolution of digital reproductions. Stimuli were presented as short clips of 3 s in order to simulate a perceptual approach toward each artwork. This methodological choice reflects evidence that while high-resolution reproductions can approximate originals in terms of resolution, scale, and color calibration, even minor deviations in texture, lighting, or saturation can influence judgments (Berns, 2001; Reymond et al., 2020). The same concern with ecological validity was applied to the measure of viewing time. Rather than relying on the estimate (by an experimenter) of the time spent in front of each work, or on the total duration of the visit, viewing time was recorded automatically in both contexts as the interval between the onset of exposure to an artwork and the initiation of the emotional response assessment, with participants free to determine how long they engaged with each artwork before responding. This yields a per-artwork engagement index comparable to the unobtrusive timing used in naturalistic observational studies (Smith and Smith, 2001). Finally, to mitigate the risk of museum fatigue, our study deliberately limited the visit to 17 artworks, balancing ecological validity with sustained attention.

## 2 Materials and methods

### 2.1 Participants

A hundred and four participants (26 males, 76 females, 1 non-binary, 1 unknown) were recruited from Université Laval (students and employees) and from the Musée national des beaux-arts du Québec (members). Participants were randomly assigned to one of two viewing context: one group viewed the original artworks at the Musée national des beaux-arts du Québec, whereas the other group viewed the digital artworks in the laboratory. All participants were French-speaking and reported normal or corrected-to-normal vision. They received CAD $10 in compensation for their participation and provided informed consent prior to taking part in the study. The research was approved by the human research ethics committee of Université Laval (approval number 2022–345/02-10-2022).

The sample was categorized into six age groups: 18–25 years (*n* = 41, 39.42%), 26–35 years (*n* = 16, 15.38%), 36–45 years (*n* = 15, 14.42%), 46–55 years (*n* = 9, 8.65%), 56–65 years (*n* = 13, 12.50%) and 66 years or older (*n* = 10, 9.62%). Regarding educational background, 4 participants (3.85%) had completed high school, 31 (29.81%) held a college diploma, 36 (34.62%) had a bachelor’s degree, 27 (25.96%) a master’s degree, and 5 (4.81%) a doctoral degree. One participant (0.96%) did not report his educational level.

### 2.2 Apparatus

Two experimental setups were used, one in the museum and one in the laboratory. In both contexts, participants reported their emotional responses using a custom-built iPad application (6th generation with a 9.7-inch retina display). In the laboratory, the digital stimuli were presented on a PC computer using E-prime 3.0 software. Participants reported the emotional response measures on the iPad positioned beside the computer monitor.

### 2.3 Stimuli

The stimuli consisted of 17 artworks, including paintings (5), glass compositions (2), ceramic objects (1), sculptures (4), photographs (1), designs (2), and mixed techniques (2), selected by curators from the Musée national des beaux-arts du Québec to elicit a variety of emotional responses. Details on the selected artworks are provided in Supplementary material S1. Selection criteria included the use of everyday materials, universal themes, cultural references, diversity of formats, and expressive use of color and gesture.

In the museum condition, participants viewed the original artworks displayed in the permanent exhibition rooms located on the second and third floors of the Pierre Lassonde Pavilion. In the laboratory condition, each artwork was presented using a standardized interface (see Figure 1). Participants first viewed a standardized 3-s 360-degree video recorded using an iPhone mounted on a handheld stabilizer to allow observation of the artwork from multiple angles. Following the video, a high-resolution still image of the artwork was displayed. The presentation interface comprised six areas: (a) the artwork title and year of production, (b) the artwork display, (c) the artist’s name, date and place of birth, together with the artwork medium, (d) a brief description of the artwork, (e) the navigation menu, and (f) the next-page button.

**Figure 1:**
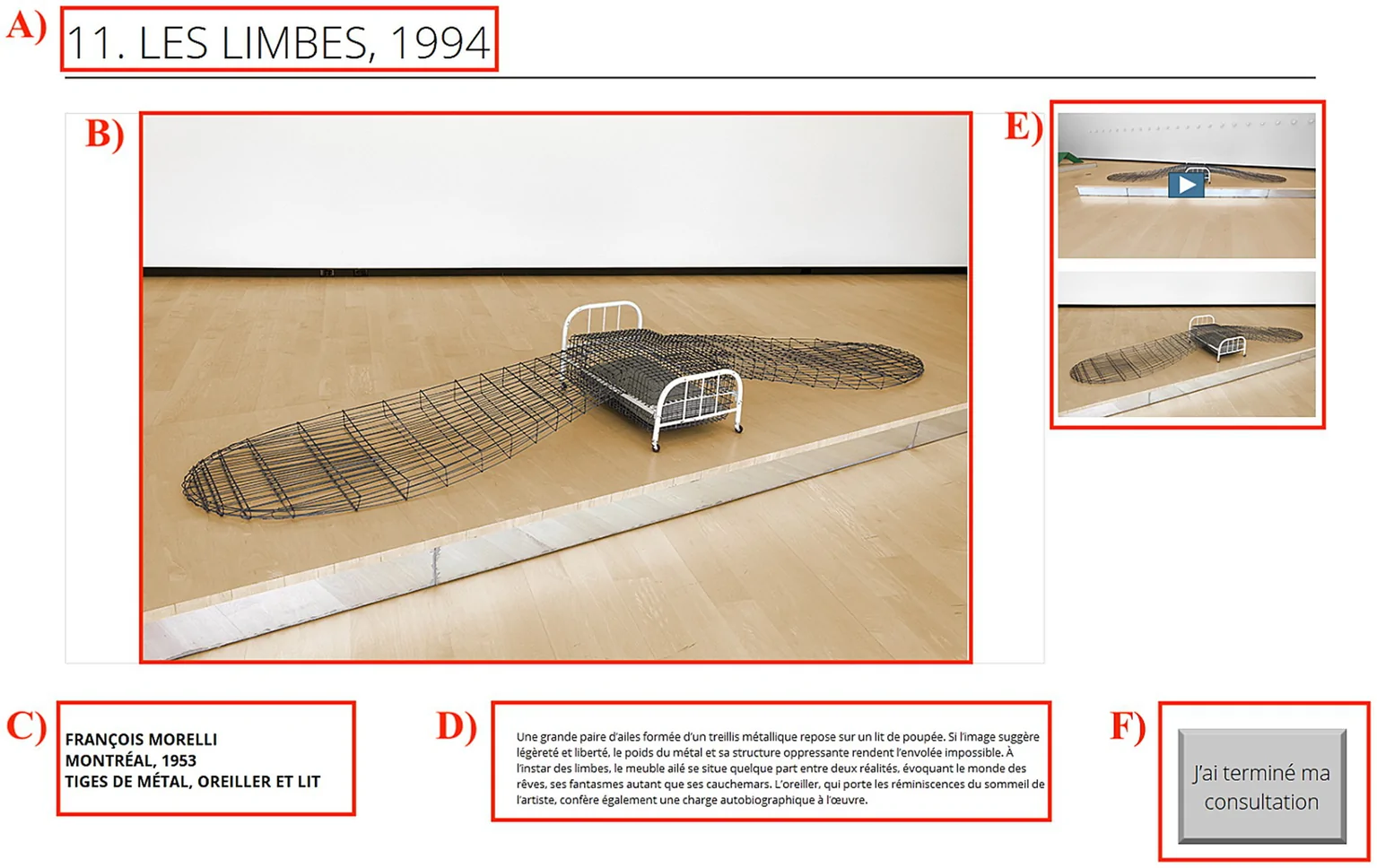
Visual interface used to present digital reproductions of artworks. The interface was divided into six areas: **(A)** the title of the artwork with the year of production, **(B)** the presentation of the artwork, **(C)** the name, date and place of birth of the artist, and the artwork’s medium **(D)** a short description of the piece, **(E)** the navigation menu, and **(F)** the next page button.

### 2.4 Measures

The same outcome measures were collected in both viewing contexts to ensure methodological consistency across experimental conditions. After viewing each artwork, participants first identified the dominant emotion they experienced by selecting one of 12 emotions displayed in a circular arrangement (see Figure 2). The 12 emotions were selected to provide broad coverage of the valence–arousal space, with three emotions representing each quadrant. The selection primarily comprised affective states described within dimensional models of emotion (Posner et al., 2005; Russell, 1980; Russell and Bullock, 1985), with fascination additionally included because of its particular relevance to aesthetic experience (Schindler et al., 2017). Emotion labels were presented to participants in French; both the original French terms and their English translations are provided in Table 1. Participants could also select neutral or other when the experienced emotion was not represented among the 12 predefined choices. No definitions of the emotion terms were provided to let participants select the label that they felt best represented their own experienced emotion. Participants then rated the valence and arousal associated with their emotional experience using two visual analog scales (Figure 3), yielding values ranging from −1 (negative/low) to 1 (positive/high). Finally, viewing time was recorded in seconds for each artwork. In both contexts, it was defined as the elapsed time between the beginning of artwork exposure and the initiation of the emotional response assessment. In the museum condition, this interval was recorded automatically by the custom-built iPad application, with timing beginning when participants indicated that they had reached the artwork. In the laboratory condition, the interval was recorded automatically by E-prime 3.0, with timing beginning at the onset of the artwork presentation.

**Figure 2:**
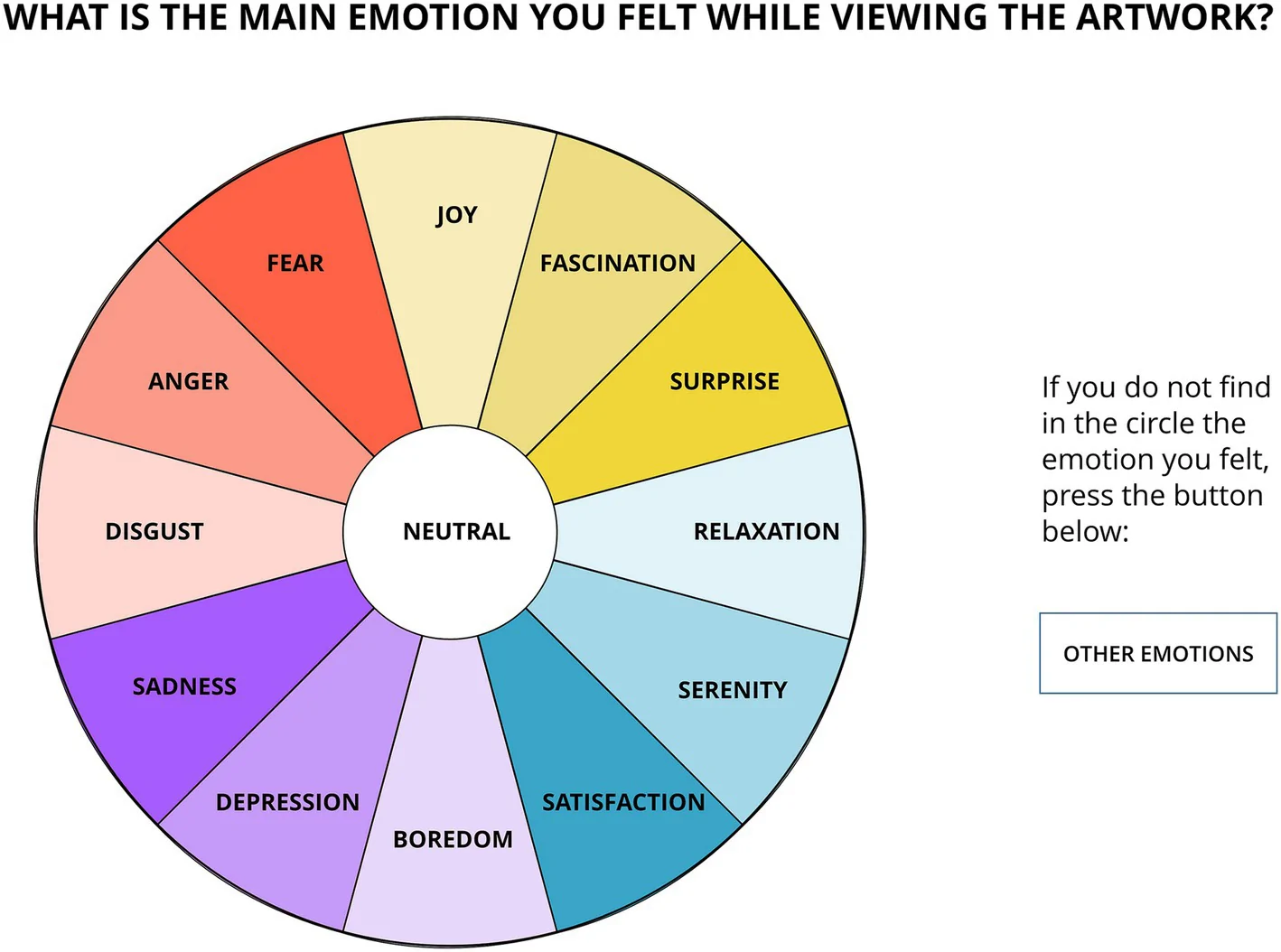
Circular visual interface designed to report the dominant emotion experienced after viewing the artwork.

**Table 1:** Arousal-valence coordinates derived from Paltoglou and Thelwall (2013).

Emotion reported (french)	English translation	Reference coordinates			Quadrant
		Ref. emotion	Arousal	Valence	
*Joie*	*Joy*	Joyous	0.13	0.95	Positive arousal/positive valence
*Fascination*	*Fascination*	Excited	0.71	0.70	Positive arousal/positive valence
*Surprise*	*Surprise*	Astonished	0.88	0.43	Positive arousal/positive valence
*Détente*	*Relaxation*	Relaxed	−0.65	0.71	Negative arousal/positive valence
*Sérénité*	*Serenity*	Serene	−0.05	0.84	Negative arousal/positive valence
*Satisfaction*	*Satisfaction*	Satisfied	−0.63	0.77	Negative arousal/positive valence
*Ennui*	*Boredom*	Bored	−0.78	−0.35	Negative arousal/negative valence
*Dépression*	*Depression*	Depressed	−0.48	−0.81	Negative arousal/negative valence
*Tristesse*	*Sadness*	Sad	−0.40	−0.81	Negative arousal/negative valence
*Dégoût*	*Disgust*	Disgusted	0.49	−0.67	Positive arousal/negative valence
*Colère*	*Anger*	Angry	0.79	−0.40	Positive arousal/negative valence
*Peur*	*Fear*	Afraid	0.79	−0.12	Positive arousal/negative valence
*Neutre*	*Neutral*	--	0.00	0.00	--

**Figure 3:**
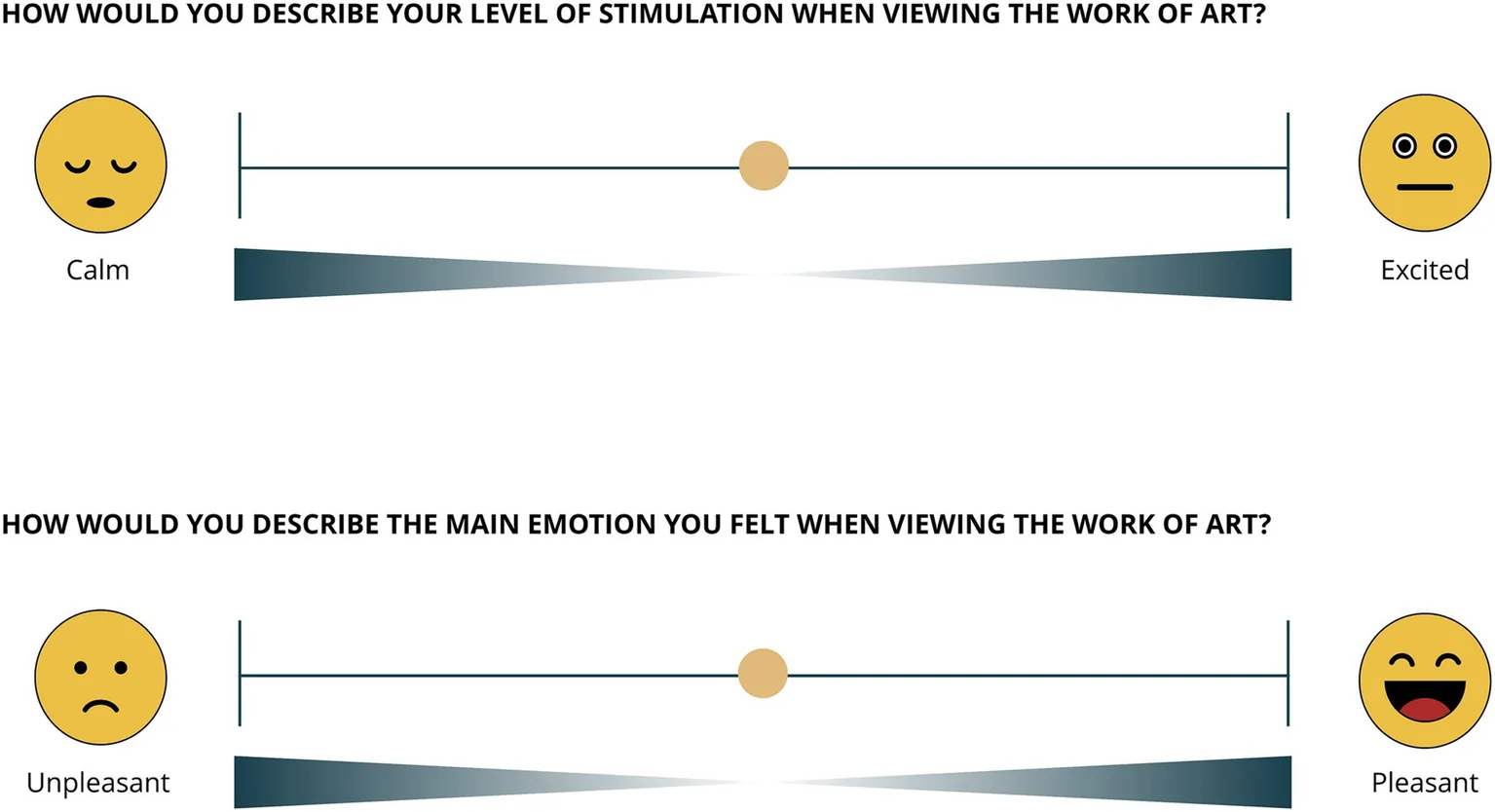
Visual analog scales designed to measure arousal (top) and valence (bottom) on a custom-built iPad application. The cursor was positioned at the center (0) and generated values from −100 to 100.

### 2.5 Procedure

#### 2.5.1 Museum condition

To optimize participants’ experience and minimize potential order effects, four distinct visiting routes were designed by varying the starting floor (second or third) and the direction of the visit (clockwise or counterclockwise). The four routes were counterbalanced across participants.

Participants completed the museum visit autonomously using the custom-built iPad application, which guided them through the assigned route and prompted them to complete the emotional response measures after viewing each artwork. A research assistant remained available to provide assistance if participants encountered technical difficulties or had difficulty locating an artwork. Participants were free to determine how long they viewed each artwork before completing the emotional response measures. On average, the museum session lasted approximately 90 min.

#### 2.5.2 Laboratory condition

Participants viewed the artworks individually while seated approximately 60 cm from the computer monitor. To ensure consistency across experimental conditions and minimize potential order effects, the artworks were presented in the same four presentation orders used in the museum condition, which were counterbalanced across participants. Participants were free to replay the video clip and view the still image for as long as they wished before reporting their emotional responses on the iPad. The laboratory session lasted approximately 60 min.

### 2.6 Design

We adopted a between-subject design in which participants were randomly assigned to one of two groups: one viewed digital reproductions of the 17 artworks at the museum, whereas the other viewed the same 17 artworks in the laboratory. Although a between-subject design may introduce greater inter-individual variability than a within-subject design, it eliminates potential effects of repeated exposure and increasing familiarity with the artworks.

## 3 Results

The emotional responses of one participant to a single artwork in the museum context were excluded from the analyses because the wrong artwork was viewed. Analyses were conducted using R (version 4.5.1) and SPSS (version 31). A two-tailed *p*-value < 0.05 was considered as statistically significant. The magnitude of correlations was interpreted according to Cohen’s (2013) guidelines, with values of 0.10, 0.30, and 0.50 representing small, medium, and large effects, respectively.

### 3.1 Correspondence with the circumplex model

To assess the extent to which the observed distribution of valence and arousal ratings reflected the theoretical distribution proposed by Russell’s (1980) circumplex model of emotional response, we compared participant-level coordinates for each artwork with their corresponding reference coordinates, derived from Paltoglou and Thelwall (2013; see Table 1). Figure 4 presents the observed and reference emotion centroids within the valence–arousal space.

**Figure 4:**
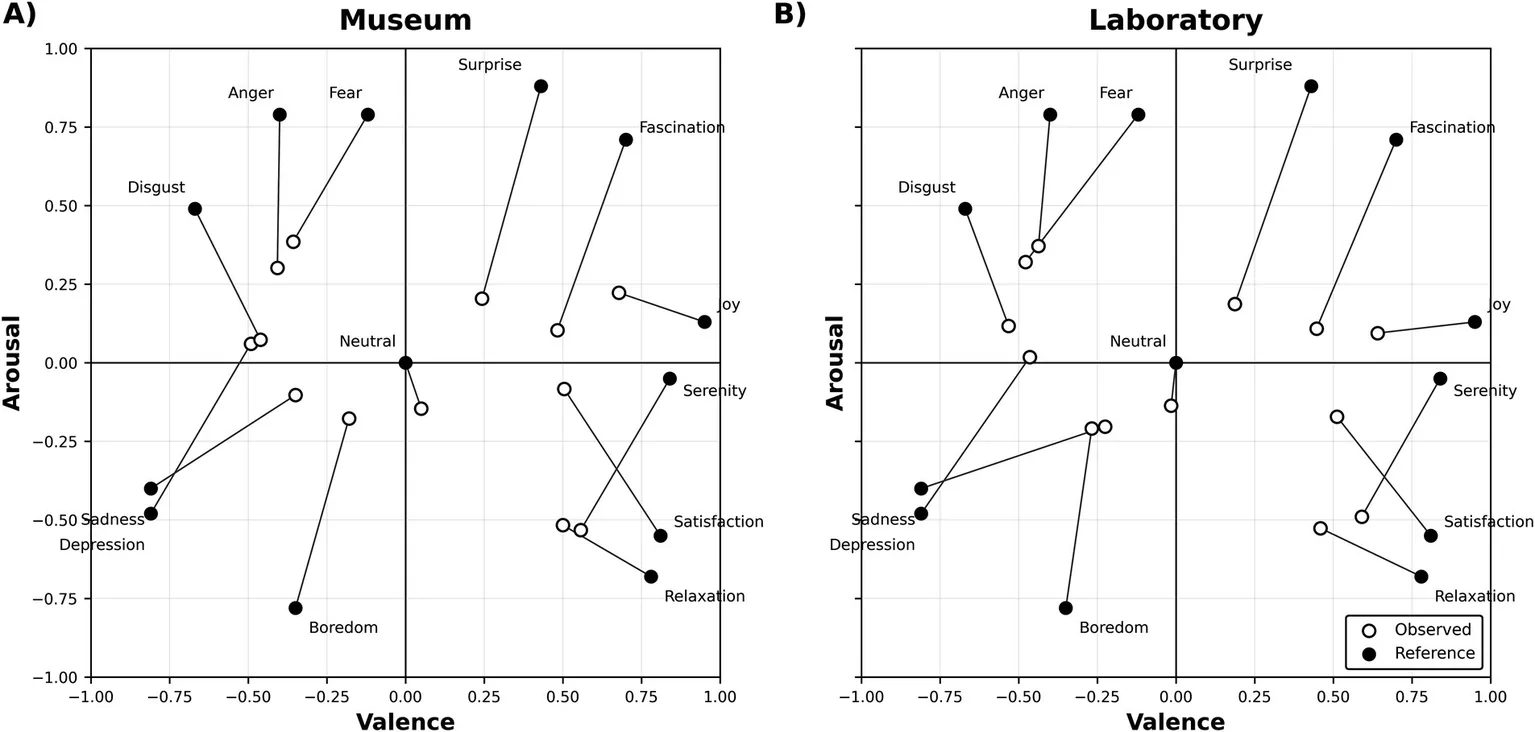
Observed and reference emotion centroids within the valence–arousal space for the museum context **(A)** and the laboratory context **(B)**.

Three complementary indices were computed to quantify the correspondence between the observed and reference affective spaces in each viewing context. These indices were used as descriptive measures of convergence with the external valence–arousal reference structure rather than as formal tests of model fit, as no established thresholds are available for determining acceptable RMSE or Procrustes disparity values in this context. For the RMSE and the Procrustes disparity, empirical reference distributions were generated by permutation. On each of 9,999 permutations, the pairing between the 13 observed emotion centroids and their reference coordinates was randomly reassigned, leaving the observed and reference configurations otherwise unchanged, and each index was recomputed. Permutations were run separately for each viewing context in R. First, the root mean square error (RMSE) quantified the average Euclidean distance between observed and reference coordinates in the two-dimensional valence–arousal space. RMSE values were identical across contexts (RMSE = 0.52 for both the laboratory and museum conditions). These values were far below the value expected under random correspondence (median permuted RMSE = 1.01 and 1.02 respectively; *p* < 0.001 in both contexts). Second, Spearman rank-order correlations were computed separately for the valence and arousal dimensions using the mean observed coordinates for each emotion (*n* = 13). For valence, correlations with the reference coordinates were strong and statistically significant in both the laboratory (*r*s = 0.88, *p* < 0.001) and museum (*r*s = 0.95, *p* < 0.001) contexts. Significant positive correlations were also observed for arousal in both the laboratory (*r*s = 0.88, *p* < 0.001) and museum (*r*s = 0.78, *p* = 0.002) contexts. Finally, a Procrustes analysis quantified the similarity of the overall spatial configurations by optimally translating, rotating, and scaling the configurations while minimizing the sum of squared distances between corresponding points (Gower, 1975). The remaining discrepancy after superimposition was quantified using the Procrustes disparity statistic (Goodall, 1991), yielding values of 0.26 for the laboratory context and 0.28 for the museum context (again far below chance with median permuted disparity = 0.90 in both contexts; *p* < 0.001).

### 3.2 The genuineness effect

#### 3.2.1 Dominant emotion ratings

Table 2 presents the frequencies of the dominant emotions reported across all artworks in the museum and laboratory contexts.

**Table 2:** Frequencies and percentages of the dominant emotion reported for all artworks by viewing context (museum vs. laboratory).

Emotion	Context	
	Museum	Laboratory
*Joy*	81 (9.2%)	65 (7.4%)
*Fascination*	167 (18.9%)	167 (18.9%)
*Surprise*	68 (7.7%)	102 (11.5%)
*Relaxation*	59 (6.7%)	42 (4.8%)
*Serenity*	57 (6.5%)	38 (4.3%)
*Satisfaction*	55 (6.2%)	46 (5.2%)
*Boredom*	68 (7.7%)	59 (6.7%)
*Depression*	22 (2.5%)	20 (2.3%)
*Sadness*	79 (8.9%)	58 (6.6%)
*Disgust*	41 (4.6%)	68 (7.7%)
*Anger*	18 (2.0%)	16 (1.8%)
*Fear*	58 (6.6%)	57 (6.4%)
*Neutral*	64 (7.2%)	86 (9.7%)
*Other*	46 (5.2%)	60 (6.8%)

#### 3.2.2 Arousal and valence ratings

Figure 5 displays the distribution of participants’ self-reported valence and arousal ratings in the two-dimensional valence–arousal space for the museum and laboratory contexts. Each bubble represents the frequency of observations at a given combination of valence and arousal values, with larger bubbles indicating higher frequencies. These dimensional ratings were reported independently of the categorical dominant-emotion response and therefore do not necessarily correspond to the theoretical location of the selected emotion in the valence–arousal space. In both contexts, many observations were concentrated near the center of the space, while additional responses extended toward both positive and negative valence as well as higher and lower arousal levels. Comparisons between the two settings suggest broadly similar patterns, with no marked differences in the overall distribution of responses across the valence–arousal space.

**Figure 5:**
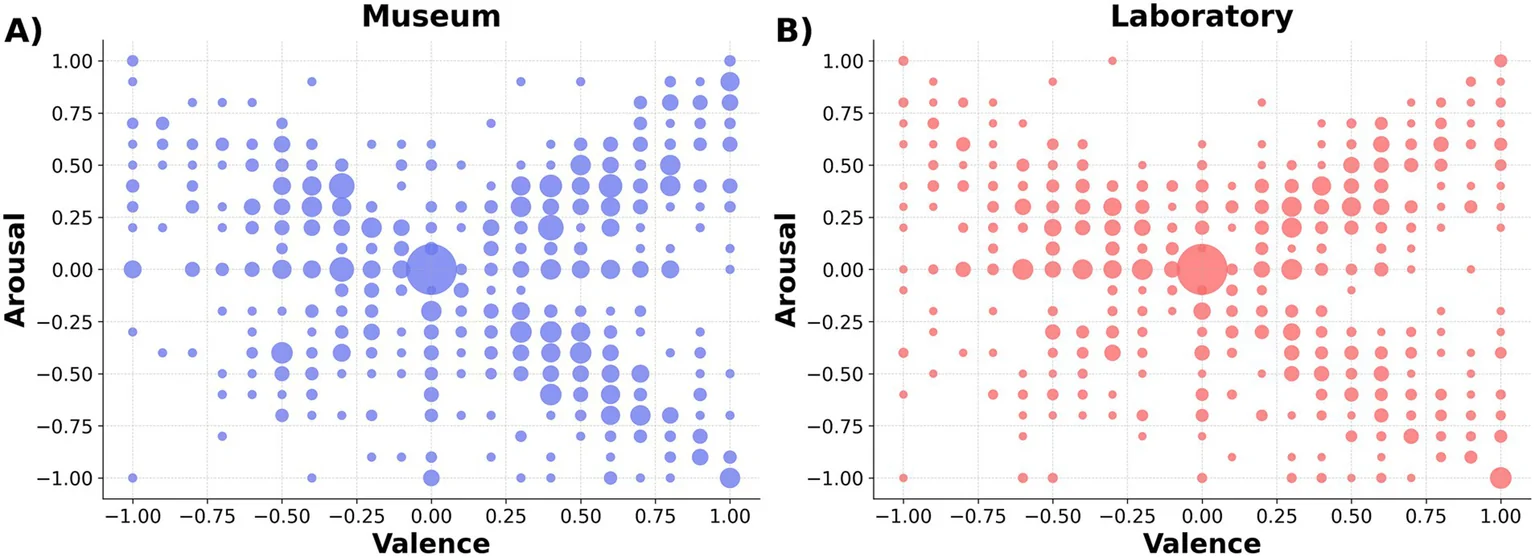
Valence and arousal ratings for all artworks in the museum context **(A)** and in the laboratory context **(B)**. Bubble size represents the frequency of observations sharing the same valence and arousal values after rounding to one decimal place.

Two separate linear mixed-effects models were fitted using restricted maximum likelihood estimation, with arousal and valence ratings as the respective dependent variables. The fixed effect was viewing context (museum vs. laboratory). Random intercepts were included for participants and artworks to account for individual differences in baseline ratings and variability between artworks. A by-artwork random slope for viewing context was also included to allow the effect of context to vary across artworks. Models were estimated using the *lmer* function from the lme4 package, with Satterthwaite’s approximation used to compute degrees of freedom and *p*-values.

Table 3 presents the mean and standard deviation of valence and arousal ratings for all artworks in the laboratory and museum contexts. Viewing context did not significantly influence either arousal ratings, *b* < −0.01, *SE* = 0.04, *t*(60.10) = −0.08, *p* = 0.94, or valence ratings, *b* = 0.06, *SE* = 0.04, *t*(32.81) = 1.49, *p* = 0.15. The arousal model explained less than 1% of the variance through the fixed effect (marginal *R*^2^ < 0.01) and 24.8% of the variance when random effects were included (conditional *R*^2^ = 0.25). Similarly, the valence model explained less than 1% of the variance through the fixed effect (marginal *R*^2^ < 0.01) and 30.1% of the variance when random effects were included (conditional *R*^2^ = 0.30).

**Table 3:** Descriptive statistics (mean and standard deviations) of arousal, valence, and viewing time (in seconds) for all artworks by viewing context (museum vs. laboratory).

Artwork	Museum			Laboratory		
	Arousal	Valence	Viewing time	Arousal	Valence	Viewing time
*Coffee set*	−0.03 (0.40)	0.26 (0.40)	51.85 (24.93)	0.05 (0.46)	0.32 (0.37)	63.29 (29.36)
*Ming X*	0.07 (0.44)	0.37 (0.43)	68.03 (36.48)	−0.02 (0.47)	0.34 (0.41)	67.53 (30.43)
*Les Petites Filles de Madame Liddell*	0.24 (0.40)	−0.30 (0.54)	71.54 (40.14)	0.09 (0.35)	−0.37 (0.45)	63.67 (26.09)
*HIM Chair*	0.03 (0.43)	0.24 (0.50)	64.97 (35.53)	0.19 (0.44)	0.06 (0.54)	64.93 (23.73)
*« Rocco » Mirror*	0.01 (0.32)	0.05 (0.40)	54.60 (28.49)	−0.07 (0.28)	−0.02 (0.36)	47.31 (19.50)
*Composition*	0.06 (0.46)	0.39 (0.44)	67.67 (32.21)	0.08 (0.45)	−0.01 (0.54)	50.85 (22.21)
*Self-Destruction*	0.23 (0.36)	−0.11 (0.49)	59.83 (33.25)	0.13 (0.45)	−0.18 (0.48)	56.58 (26.96)
*Reflecting Bear*	−0.26 (0.52)	0.56 (0.30)	49.87 (27.00)	−0.28 (0.46)	0.56 (0.32)	54.72 (25.04)
*Brome Lake*	−0.04 (0.47)	0.39 (0.41)	71.28 (31.19)	−0.14 (0.44)	0.46 (0.42)	74.56 (31.34)
*Kanaka*	0.16 (0.48)	0.43 (0.41)	65.79 (26.01)	0.15 (0.51)	0.31 (0.46)	67.10 (30.31)
*Limbo*	−0.05 (0.41)	−0.10 (0.53)	64.41 (32.35)	0.08 (0.37)	−0.07 (0.51)	65.44 (26.83)
*Tarpaulin No. 1*	−0.18 (0.39)	0.15 (0.41)	61.54 (28.93)	−0.14 (0.40)	−0.07 (0.45)	62.66 (30.40)
*Déprises II*	0.18 (0.43)	−0.27 (0.54)	70.71 (30.93)	0.17 (0.38)	−0.28 (0.48)	68.42 (26.83)
*The Arborescent Heart*	−0.40 (0.43)	0.43 (0.41)	66.60 (29.80)	−0.23 (0.48)	0.38 (0.47)	65.83 (26.08)
*Tornado*	0.17 (0.39)	−0.03 (0.47)	68.68 (27.95)	0.25 (0.30)	−0.01 (0.50)	69.91 (25.32)
*The Scar Project*	0.01 (0.39)	−0.10 (0.53)	95.72 (39.38)	−0.06 (0.44)	0.12 (0.46)	78.43 (31.17)
*After Nature I*	−0.33 (0.50)	0.29 (0.47)	58.89 (27.11)	−0.31 (0.41)	0.07 (0.43)	66.37 (22.52)

### 3.3 Viewing time

Due to technical issues during data collection at the museum, 0.68% (12/1767) of the viewing time data were unavailable and were excluded from the analyses. In addition, 0.68% (12/1755) of the remaining observations were identified as outliers using Tukey’s method (i.e., observations located more than three times the interquartile range beyond the first and third quartiles) and were excluded from subsequent analyses.

Table 3 summarizes the mean viewing times (with standard deviations) for all artworks in the museum and laboratory contexts. A linear mixed-effects model was conducted with viewing time as the dependent variable. Viewing context (museum vs. laboratory) was included as the fixed effect. Random intercepts were specified for participants and artworks, along with a by-artwork random slope for viewing context to allow the effect of context to vary across artworks. Viewing context did not significantly influence viewing time, *b* = 1.43, *SE* = 4.56, *t*(106.16) = 0.31, *p* = 0.75. The model explained less than 1% of the variance through the fixed effect (marginal *R*^2^ < 0.01) and 55.6% of the variance when random effects were included (conditional *R*^2^ = 0.56).

## 4 Discussion

The present study was designed to examine two main questions: (1) how well the circumplex model describes emotional responses to artworks, both in terms of the correspondence between reported emotions and their reference coordinates and in terms of the coverage of the emotion vocabulary derived from the model, and (2) whether genuine artworks viewed *in situ* elicit stronger and more polarized emotional responses than their digital reproductions. Our goal was to further investigate the impact of encountering originals *in situ* versus their digital counterparts.

### 4.1 The nature and process of emotions

Our findings on the correspondence between observed emotional responses and the valence–arousal organization described by dimensional models, such as the circumplex model, are relevant to how emotions are constructed and reported (see Russell and Barrett, 1999). Although the observed affective space was more compressed than the external reference structure, the relative organization of emotions along the valence and arousal dimensions corresponded substantially to the reference coordinates. Compression of this kind is characteristic of visual analog scales, on which responses tend to be drawn toward the midpoint. Ratings of valence and arousal showed strong alignment with Russell’s circumplex structure, particularly for valence. Arousal is the dimension for which self-report is least straightforward. The weaker fit to arousal may reflect a property of the dimension itself rather than a failure of the model in an aesthetic context. This outcome is in line with evidence that visitors find arousal particularly difficult to evaluate after viewing artwork (Schmitt et al., 2018), and with the argument that arousal cannot be simply equated with the intensity of valence (Kuppens et al., 2013).

These findings also connect to the debate over the nature and role of aesthetic emotions. Menninghaus et al. (2019, 2020) argue that certain emotions, such as awe, being moved, and fascination, can play a distinctive role in aesthetic evaluation and appreciation, without necessarily implying that these emotions depend on distinct underlying mechanisms. Skov and Nadal (2020), in contrast, question the need to posit aesthetic emotions as a distinct class and emphasize continuity between emotional responses to art and those elicited in other domains (see also Fingerhut and Prinz, 2020).

A second set of findings speaks to the coverage of the emotion labels available within these models. The labels we derived from dimensional models did not fully capture the range of emotional responses elicited by art. Fascination emerged as the most frequently reported emotional response. In addition, 7% of responses were in the “other” category rather than any of the predefined emotion labels. The prominence of fascination, combined with the responses assigned to Other, is consistent with the resolution limit identified in data-driven mappings of emotional experience (Stamkou et al., 2024): a 12-point lexicon derived from core affect necessarily forces states such as being moved, nostalgia, or mysteriousness onto the nearest available label. At the same time, participants seem to draw on the full range of predefined categories, including both positive and negative everyday emotions (see Table 2), and the organization of responses within valence–arousal space corresponded closely to the theoretical configuration. These two patterns of results provide evidence that emotional responses to art share a common dimensional representational space with emotions in other domains, even though the categories defined within that space are limited in resolution (see also Cowen and Keltner, 2017).

Beyond the nature of emotion, the results of the present study are informative on process theories of emotion. Scherer and Moors (2019) conceptualize emotions as emerging from dynamic, multi-level processes, unfolding through recursive appraisals and closing in a subjective labeling decision (see also Moors, 2014). Teoh et al. (2023) propose that self-reports of emotions reflect decision-like processes of evidence accumulation. The modest categorical agreement and high variability could be indicative of these processes taking place when participants were required to settle on specific labels. According to the Vienna Integrated Model of Art Perception (Pelowski et al., 2017b), emotional responses arise at multiple stages of processing. Initial perceptual processing (within a few hundred milliseconds) produces primary affective responses in terms of valence and arousal, consistent with appraisal theories that highlight novelty and so-called pleasantness checks (Scherer and Moors, 2019). The intrinsic pleasantness check is described as an early appraisal mechanism that rapidly assigns positive or negative valence to a stimulus based on perceptual fluency and sensory qualities. Later stages involve integration and sensemaking, where viewers attempt to match the artwork with prior patterns and schemas and generate more differentiated emotions (e.g., sadness, joy, fascination). These reflective processes are more open to modulation by context and expertise. Pelowski et al. (2025) show that such top-down (re)classifications can occur after several seconds of viewing. Our measures fall within this later window: participants reported a dominant emotion label and rated valence and arousal after a self-paced viewing episode lasting on average between 47 and 78 s, so they reflect these later stages rather than the initial affective response. If genuineness were a salient contextual cue, we might expect it to influence later stages of appraisal. The VIMAP would predict little or no genuineness effect on early emotional responses, but potential differences in later reflective appraisals. Our results, however, showed no reliable genuineness effect on self-reported valence or arousal, even though our measures were collected after extended museum viewing.

### 4.2 The genuineness effect

Our results provide no evidence of a genuineness effect on emotional responses. Arousal ratings did not differ reliably between the museum and the laboratory, nor did valence ratings, and viewing context accounted for approximately 1% of the variance in each model. Considerably more variance was associated with differences between participants and between artworks: emotional responses varied substantially from one viewer to another and from one artwork to another.

Using an experimental design tailored to dissociate genuineness and physical context, Brieber et al. (2015a) found no independent effects of either factor. Pelowski et al. (2017a) have argued that multiple levels (artwork, viewer, and museum environment) contribute to constructing aesthetic experiences (see also Pelowski and Specker, 2020). Our design, by embracing this combination, captures the reality of art encounters: genuineness is experienced as the synergy of original artworks with their contextual framing. As argued by Tinio and Specker (2023), museums may elevate aesthetic experience through the combined influence of genuineness, curatorial authority, and context.

### 4.3 Viewing time

In terms of viewing behavior, participants did not spend significantly longer with artworks in the museum than in the laboratory. Viewing time was recorded automatically in both contexts as the interval preceding the emotional response assessment (see Brieber et al., 2014; Smith and Smith, 2001). One reason for this null result may be that a label was available in both conditions. In the museum, participants could read the label accompanying each work; in the laboratory, the interface displayed a label with the same information as the wall label in the museum (the title, year, artist, medium, and a brief description) alongside the artwork. Because our measure extended over the total interval preceding the emotional response assessment, it included time spent reading the label. Labels are ordinarily part of the museum experience, and although only about half of visitors read them (Smith et al., 2017), presenting the same information in both contexts may nonetheless have removed a source of difference that would otherwise favor the museum.

As mentioned, mean viewing times in our study ranged from approximately 47–78 s across artworks and contexts. These durations contrast with the classic study of Smith and Smith (2001), which documented a median viewing time of only 17 s for masterpieces at the Metropolitan Museum of Art, with more than 90% of visitors spending less than 1 min per artwork. However, it is important to note that Smith and Smith’s observations were made under fully naturalistic conditions, whereas our participants were explicitly asked to report their emotional responses, a task that likely fostered deeper engagement. The divergence between our results and the brief viewing times reported in naturalistic studies (Smith and Smith, 2001) suggests that asking participants to articulate their emotional responses may itself function as a reflective task that deepens engagement. This interpretation aligns with models of aesthetic experience that encapsulate later stages of reflective evaluation (including the explicit labeling of emotions) serve to prolong attention and enhance interaction with artworks (Leder et al., 2004; Pelowski et al., 2017a). In this sense, our methodological design may be taken to highlight how the very act of reporting emotions can become part of the aesthetic process, supporting the combined impact of genuineness and context.

### 4.4 Methodological and practical implications

From a methodological perspective, our study contributes to a growing consensus that ecologically valid, mixed-method approaches should become standard practice in museum research (e.g., Zhang et al., 2023). Combining user-friendly valence–arousal sliders that enable continuous reporting of affective state (Betella and Verschure, 2016), brief categorical self-reports, and metrics of emotional variety across visits offer a more complete evaluation of affective experience and its trajectories. Such approaches can reveal outcomes that matter most for cultural institutions (e.g., reflection, meaning, and well-being) rather than relying solely on global ratings of liking or duration of presence in a museum. An additional line of evidence points to the value of multimodal measures that extend beyond self-report. Kühnapfel et al. (2024) showed that bodily movement patterns can serve as markers of art experience that seem to differentiate between moments of insight, arousal, and positive affect. Incorporating such embodied signatures with eye-tracking, physiological measures, and subjective ratings could yield a richer account of aesthetic experience and emotional response.

Some observations from the current study point to some potential practical implications. Our results support the idea that museums could design for emotional diversity at the level of the visit. Rather than focusing only on how individual artworks are liked, museums could measure the variety of emotions across an entire visit (Rodriguez et al., 2021; Tröndle et al., 2014). One suggestion is that curatorial strategies might be oriented toward diversifying emotional trajectories (see Pelowski et al., 2017b), for example, by alternating experiences of awe, curiosity, and calm, rather than amplifying a single dominant register. Asking visitors to articulate their emotional responses may extend engagement: viewing times in both of our contexts were substantially longer than those documented under naturalistic conditions (Smith and Smith, 2001), although this comparison is across studies. However, such interventions are not neutral: prompts change behavior and should be framed as part of the design of the museum experience itself.

Another implication concerns the growing use of digital affective interventions. Interactive tools such as narrative audio or “emotional journey” can scaffold engagement and extend reflection beyond the gallery (Benford et al., 2022). Yet visitors may be prone to over-trusting machine-generated “emotion readouts,” adjusting their self-understandings to align with what the system displays. This highlights the need for transparency and interpretive framing, in which AI and affective computing are positioned as companions to visitor reflection rather than as diagnostic authorities (see Varutti, 2023). Such concerns can be found in practitioner surveys that advocate the importance of a human-centric approach to AI in museums, where curatorial oversight is central (Derda and Predescu, 2025). Emotion should be treated simultaneously as an outcome to cultivate (through diversity, depth, and meaningfulness) and as a process to support through reflection and dialogue. By aligning spatial design, curatorial narratives, digital mediation, and mixed-methods evaluation, museums can design for varied, reflective, and emotionally meaningful experiences that visitors increasingly expect. Our study complements emerging perspectives in museology by showing how emotional responses to artwork can inform the evolving practices of museum design and visitor-centered creation (Varutti, 2023).

### 4.5 Limitations and future work

Some limitations of the present study should be acknowledged. First, our design contrasted genuine artworks in a museum with digital reproductions in a laboratory-like setting. This ecological, but confounded, comparison reflects how artworks are typically encountered in everyday contexts (originals in museums and reproductions online) but does not allow disentangling the independent contributions of genuineness and context. Future work could combine this approach with factorial designs that systematically vary genuineness to clarify their respective and interactive effects (e.g., Grüner et al., 2019) using emotional response and viewing time as dependent variables.

Although our study measured emotions more directly than many previous investigations, we relied primarily on self-reports of valence, arousal, and categorical emotion labels. As Walle and Dukes (2023) note, valence is often conceived as a bipolar construct that may oversimplify emotions into “positive” and “negative” categories. Although physiological measures can provide indicators of emotional arousal or activation (e.g., electrodermal activity, heart rate variability; Cittadini et al., 2023), physiological assessment of valence is less straightforward. Facial electromyography (fEMG), for example, can provide physiological indices associated with affective valence through patterns of facial muscle activity (e.g., Gerger et al., 2014), although such measures remain indirect and are generally most informative when considered alongside self-report and other measures. This brings up the importance of adopting a multimodal approach by combining subjective ratings with data from physiology and behavior (see Kühnapfel et al., 2024). Tschacher et al. (2012) obtained significant associations between physiological responses and five appraisal dimensions derived from visitor ratings (Aesthetic Quality, Surprise/Humor, Negative Emotion, Dominance, and Curatorial Quality), which was taken to indicate that heart rate variability and skin conductance in museums are linked to aesthetic-emotional evaluations.

Because no explicit definitions of the emotion terms were provided, individual differences in their interpretation may have contributed to variability in categorical emotion responses. In addition, although fascination was included because of its particular relevance to aesthetic experience, the predefined set did not include other aesthetic emotions such as awe or being moved. The fact that 7% of responses were classified as “Other” suggests that the predefined categories may not have captured the full range of emotional experiences elicited by the artworks. Our procedure also required participants to select a single dominant emotion, even though emotional responses to art are often dynamic, layered, and even co-activated (Menninghaus et al., 2019; Scherer, 2005; Scherer and Moors, 2019). Aesthetic emotions in particular may involve blends of positive and negative affect (see Larsen et al., 2017), such as the simultaneous experience of sadness and pleasure (Menninghaus et al., 2020). Future research should therefore consider a broader range of aesthetic emotion categories and approaches that allow participants to capture the fluidity and multi-layered nature of art-elicited emotions (Schindler et al., 2017).

## 5 Conclusion

Regarding the nature of aesthetic emotions, our results can be taken to suggest that aesthetic emotions may be best understood not as categorically separate from everyday emotions, but as based on common dimensional structures while exhibiting phenomenological signatures shaped by aesthetic context and appraisal processes (see Scherer and Moors, 2019). The dimensional structure seems relatively adequate (as valence and arousal ratings do map onto the theoretical configuration in both contexts), while the categorical vocabulary conventionally built upon that structure does not fully cover what viewers report. Whether these emotions also play a key role in aesthetic evaluation, as posited by Menninghaus et al. (2019, 2020), is a further question that the present design, which did not include liking or beauty judgments, cannot address.

Beyond conceptual contributions, one implication of this study lies in its methodological approach. This work contributes to the broader movement toward multimodal emotion assessment in art research (Tröndle et al., 2022). Future research may benefit from adopting similar mixed-method approaches that combine self-report, behavioral, and physiological measures to build a more complete picture of aesthetic experience (Specker, 2025). That viewing original artworks in the museum and reproductions in the lab elicited similar levels of arousal and valence lends further support to the idea that the genuineness effect is either absent or very small. Even if valence and arousal ratings did not differ significantly between genuine and digital experience, having participants reflect on their emotional response to artwork may foster sustained attention and deep engagement (Brieber et al., 2014).

## Data Availability

The raw data supporting the conclusions of this article will be made available by the authors, without undue reservation.
