# Supplementary material for: Emotional responses to art in situ and on screen: a test of the circumplex model and the genuineness effect
Source: Front Psychol. 2026 Oct 8;17:1960728. doi: 10.3389/fpsyg.2026.1960728 (PMC13686909; doi:10.3389/fpsyg.2026.1960728)
Supplement: Supplementary file 1 [file Data_Sheet_1.PDF]

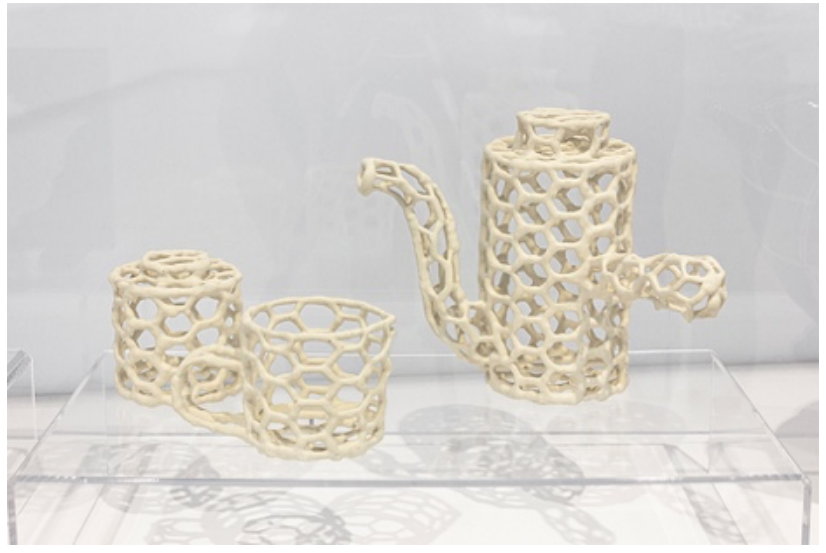

Category: Ceramics
Léopold L. Foulem
Coffee Set, 1988
White earthenware and wire-mesh
MNNAQ, purchase (1997.103.01-02-03)
© Succession Léopold L. Foulem

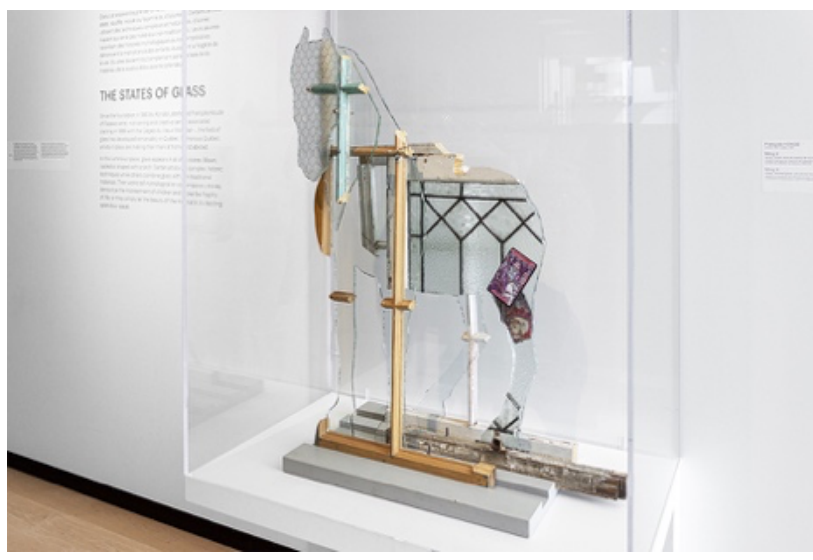

Category: Glass
François Houdé
Ming X, 1986
103.5 x 75.6 x 38 cm
Glass, stained glass, wood and tarot card
MNBAQ, purchased for the Prêt d'oeuvres d'art collection in 1988, transferred to the permanent collection (1994.232)
© Succession François Houdé

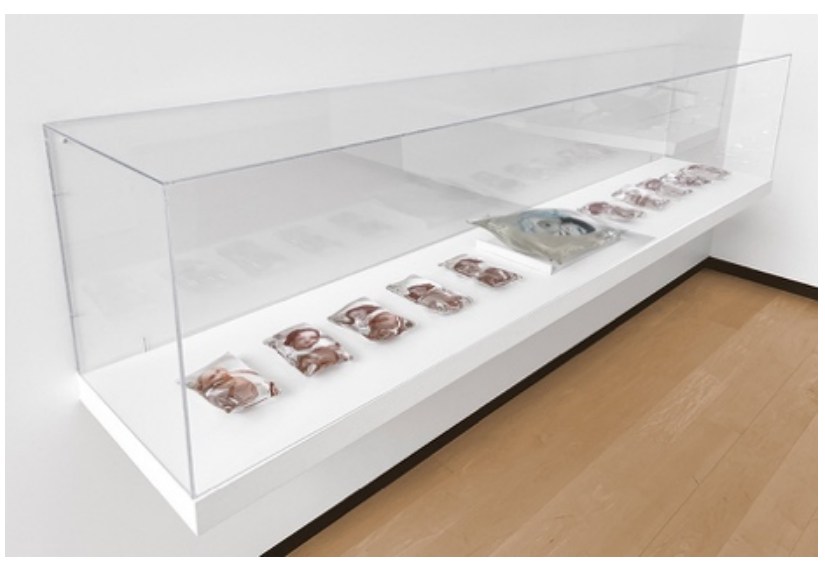

Category: Glass
Michèle Lapointe
Les Petites Filles de Madame Liddell, from the « Contes muets », 2006
7.6 x 210 x 31 cm (ensemble)
Solid glass, decalcomania fusion using iron oxide, photography, wood and collage
MNBAQ, gift of the artist (2013.320)
© Michèle Lapointe

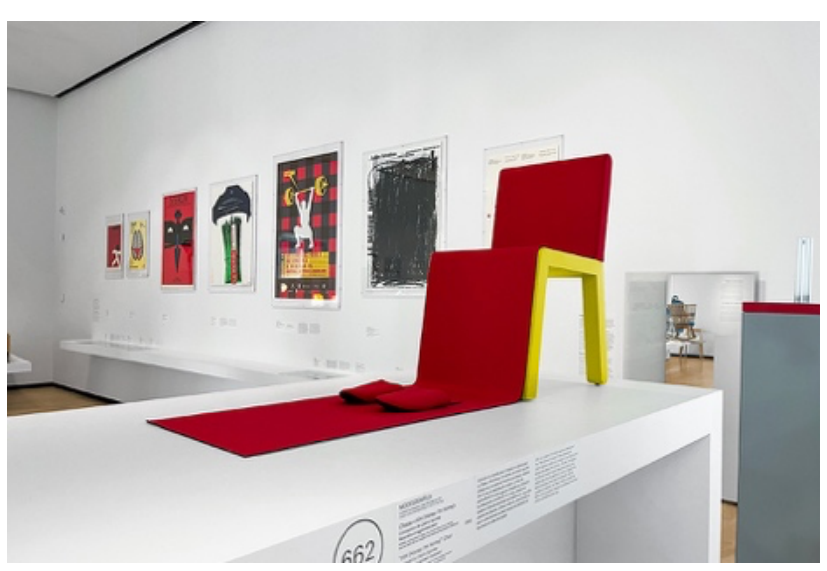

Category: Design
Modesdemploi, design by Cédric Sportes
HIH ("Honey I'm Home") Chair, 2002
76 x 41 x 137 cm (rug); 76 x 41 x 50 cm (chair)
Neoprene and painted chipboard
MNBAQ, purchased with the support of the Canada Council under its Acquisition Assistance Program (2006.231)
© Modesemploi

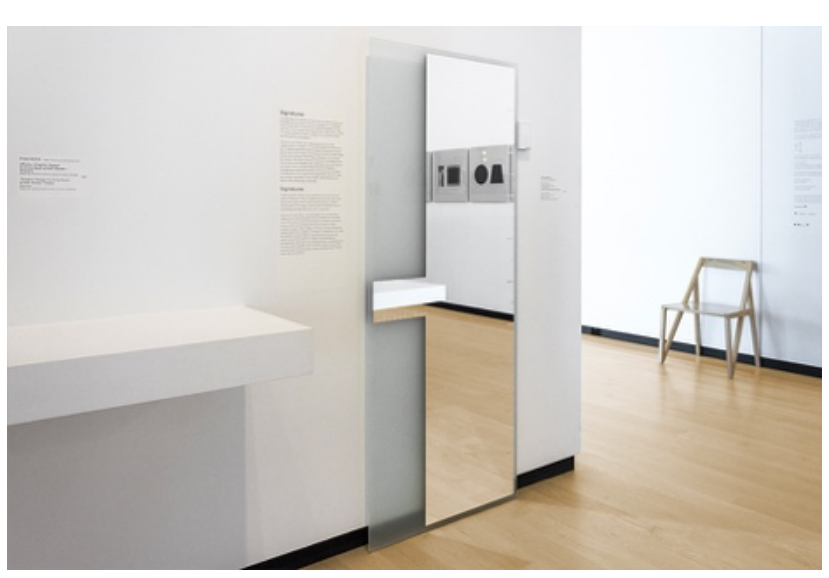

Category: Design
Modesdemploi, design by Cédric Sportes
« *Rocco* » *Mirror,* 2002
183.5 x 76 cm
Glass and chipboard
MNBAQ, purchased with the support of the Canada Council under its Acquisition Assistance Program (2006.232)
© Modesemploi

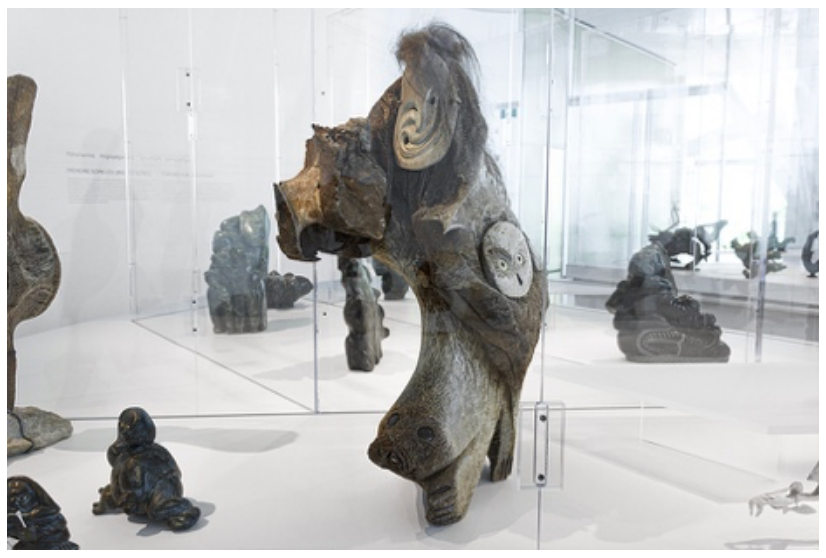

Category: Sculpture
Manasie Akpaliapik
Composition, 2001
84 x 61 x 37 cm
Whalebone, musk-ox fur, caribou antler, baleen
MNBAQ, purchase (2007.25)
© Manasie Akpaliapik

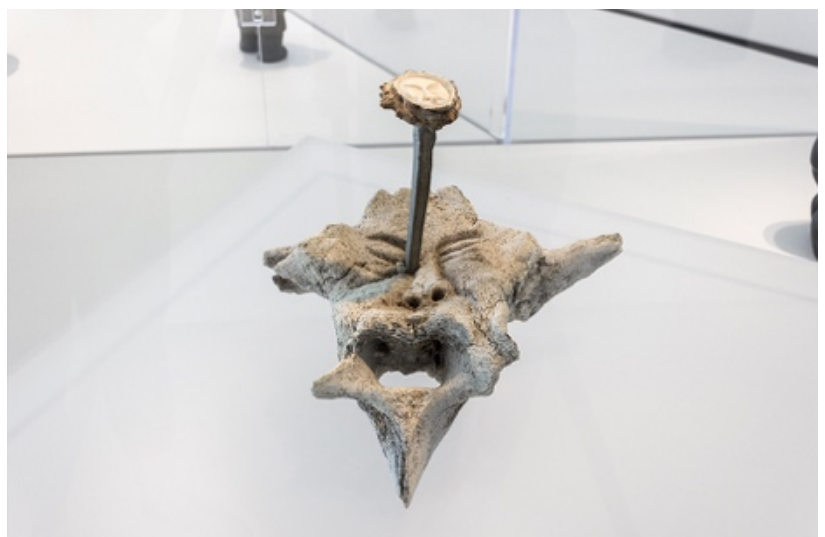

Category: Sculpture
Manasie Akpaliapik
Self-Destruction, 1995
29.6 x 35.1 x 33.1 cm
Whalebone, stone and caribou antler
MNBAQ, Brousseau Inuit Art Collection, gift of Galerie Brousseau et Brousseau inc (2005.490)
© Manasie Akpaliapik

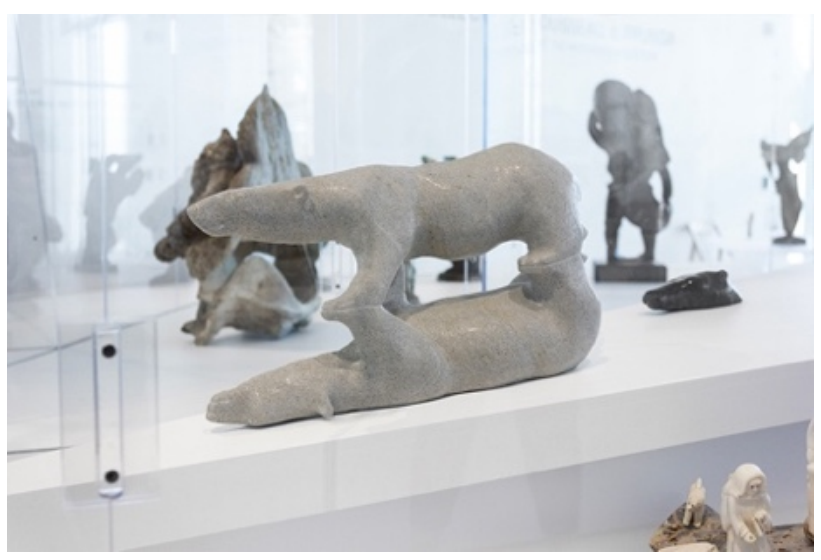

Category: Sculpture
Pauloosie Tukpanie
Reflecting Bear, 2006
23.5 x 45.5 x 14.5 cm
MNBAQ, gift of the Galerie Brousseau and Brousseau inc (2007.28)
© Pauloosie Tukpanie

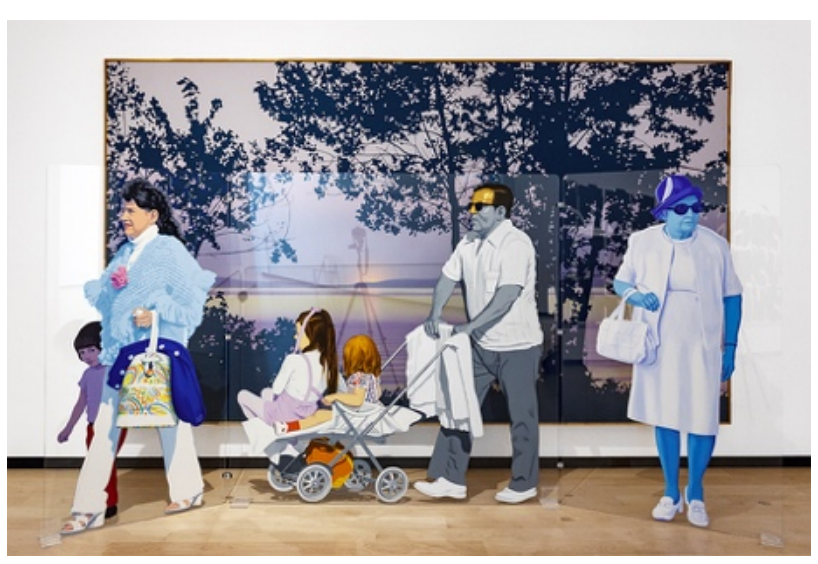

Category: Painting
Edmund Alleyn
Brome Lake, from « *La Suite québécoise* », 1973-1974
211 x 366 cm (canvas); 173 x 310 cm (panels)
Acrylic on canvas and Plexiglass panel
MNBAQ, purchase (2007.18)
© Succession Edmund Alleyn

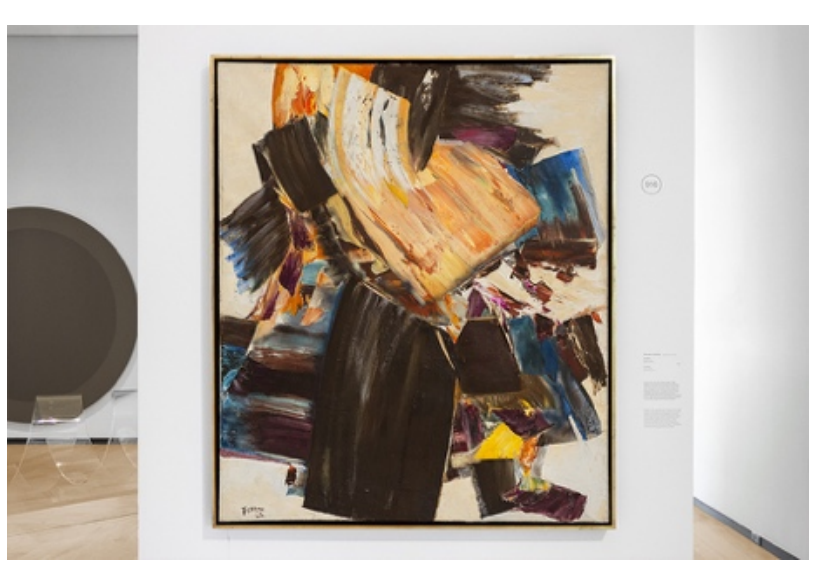

Category: Painting
Marcelle Ferron
Kanaka, 1962
201.5 x 171 cm
Oil on canvas
MNBAQ, purchase (1971.74)
@ Succession Marcelle Ferron / CARCC, Ottawa

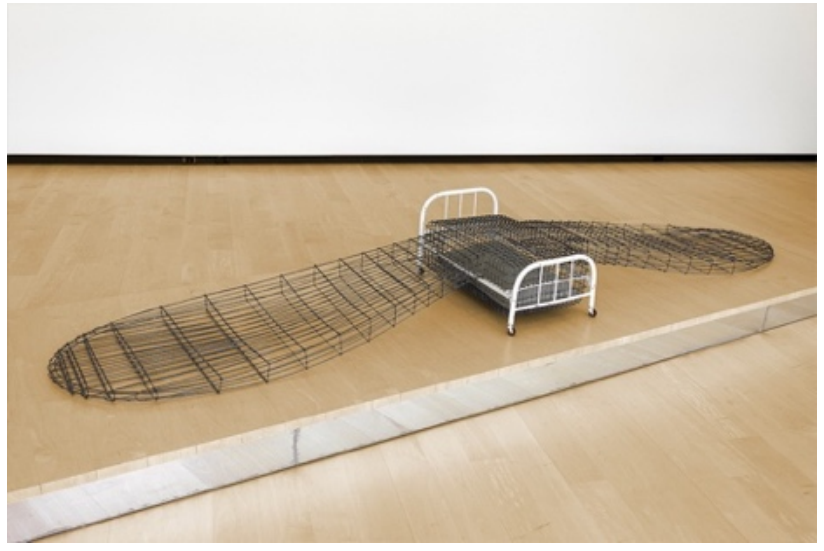

Category: Sculpture
François Morelli
Limbo, 1994
40 x 377 x 74 cm
Metal rod, pillow and bed
MNBAQ, purchase of the Prêt d'oeuvres d'art collection, 1999, transfer to the permanent collection (2005.2758)
@ François Morelli

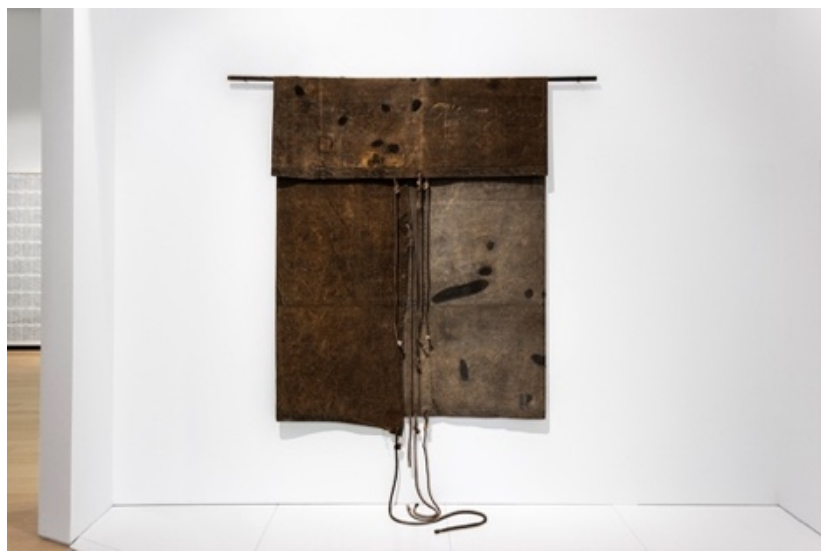

Category: Sculpture
Betty Goodwin
Tarpaulin No. 1, 1974
245 x 203 cm
Gesso on tarpaulin, rope
MNBAQ, purchase (2006.36)
@ Betty Goodwin

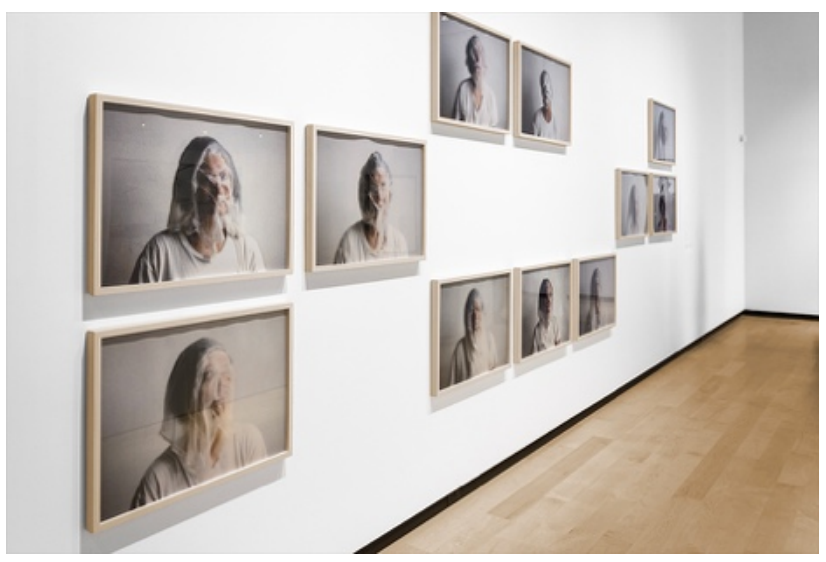

Category: Photography
Nicole Jolicoeur
Déprises II, 1999
191 x 613 cm (whole)
Digitized Polaroids, ½
MNBAQ, purchase with the support of the Canada Council for the Arts Acquisition Assistance Program (2008.03)
@ Nicole Jolicoeur

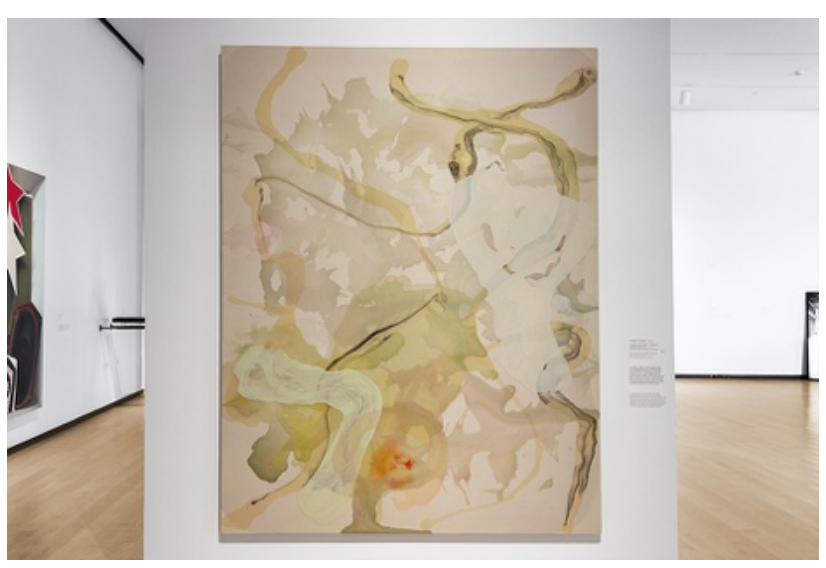

Category: Painting
François Lacasse
The Arborescent Heart / Restrain, 2002
228.5 x 178 cm
Acrylic and ink on canvas
MNBAQ, purchase with the support of the Canada Council for the Arts Acquisition Assistance Program (2004.414)
@ François Lacasse

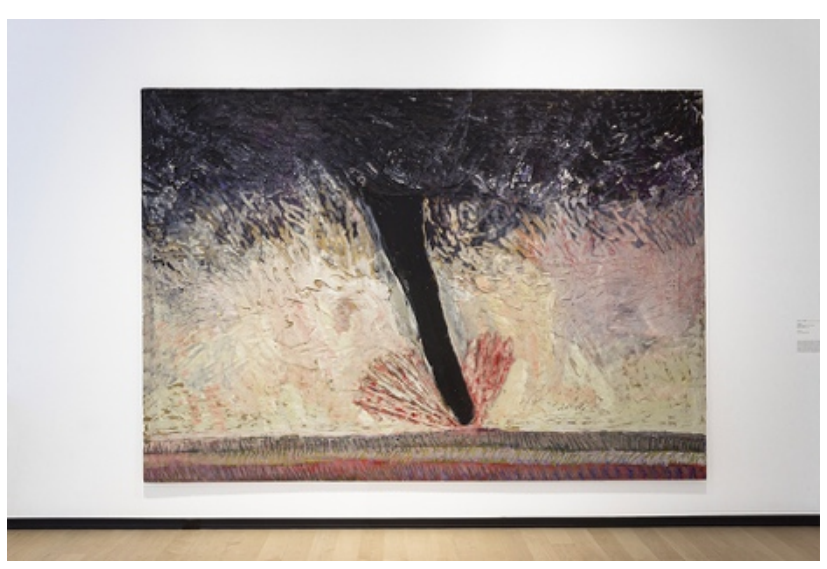

Category: Painting
Paterson Ewen
Tornado, 1989
244 x 350.5 cm
Acrylic on gouged plywood
MNBAQ, gift of Paul Mailhot (2004.430)
@ Mary Alison Handford

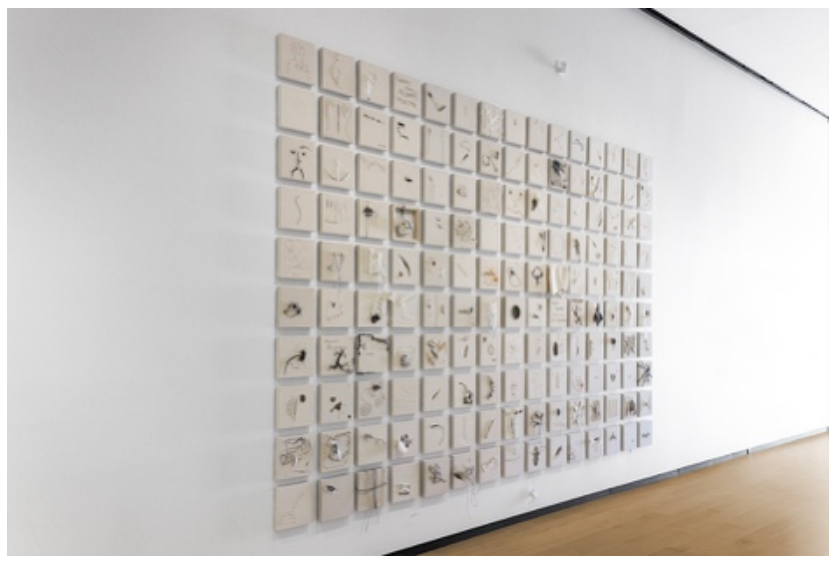

Category: Mixed media
Nadia Myre
The Scar Project, 2005-2013
25.5 x 25.5 cm (each element)
String, wool, thread and nails on canvas with perforations
MNBAQ, purchase with the support of the Canada Council for the Arts Acquisition Assistance Program (2014.140)
@ Nadia Myre / CARCC, Ottawa

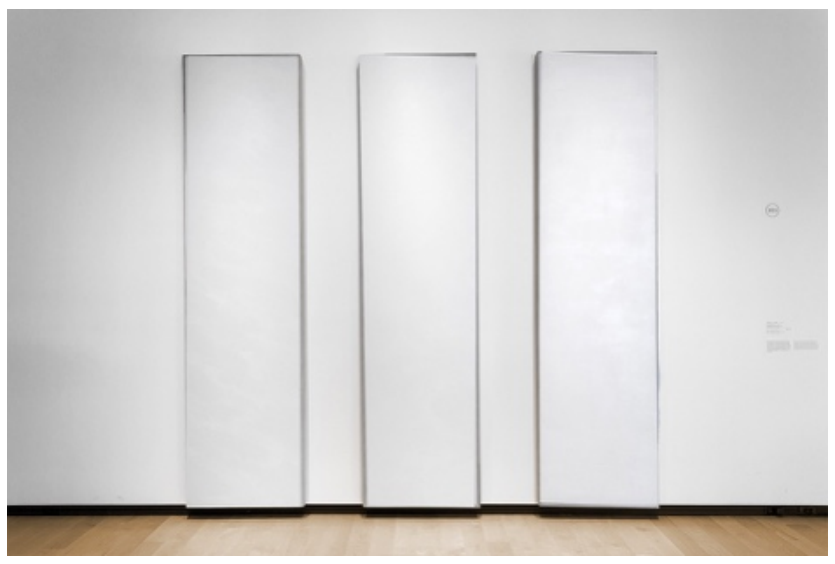

Category: Painting
Stéphane La Rue
After Nature 1, 2000-2001
303.5 x 79 cm (each)
Acrylic on canvas mounted on box
MNBAQ, purchase (2002.04.01-02-03)
@ Stéphane La Rue
